# Global trends in tumor microenvironment-related research on tumor vaccine: a review and bibliometric analysis

**DOI:** 10.3389/fimmu.2024.1341596

**Published:** 2024-02-06

**Authors:** Ying Liu, Sixin Li, Lu Chen, Lin Lin, Caijuan Xu, Huiwen Qiu, Xinyu Li, Hui Cao, Kun Liu

**Affiliations:** ^1^ Department of Psychiatry, The School of Clinical Medicine, Hunan University of Chinese Medicine, Changsha, Hunan, China; ^2^ Department of Psychiatry, Brain Hospital of Hunan Province (The Second People’s Hospital of Hunan Province), Changsha, Hunan, China; ^3^ Department of Gastroenterology, The School of Clinical Medicine, Hunan University of Chinese Medicine, Changsha, Hunan, China; ^4^ Department of Gastroenterology, Brain Hospital of Hunan Province (The Second People’s Hospital of Hunan Province), Changsha, Hunan, China; ^5^ Scientific Research Management Department, Brain Hospital of Hunan Province, The Second People’s Hospital of Hunan Province, Changsha, Hunan, China; ^6^ Department of Neurosurgery, The School of Clinical Medicine, Hunan University of Chinese Medicine, Changsha, Hunan, China; ^7^ Department of Neurosurgery, Brain Hospital of Hunan Province (The Second People’s Hospital of Hunan Province), Changsha, Hunan, China

**Keywords:** tumor vaccines, tumor microenvironment, bibliometric analysis, hotspots, CiteSpace, VOSviewer

## Abstract

**Background:**

Tumor vaccines have become crucial in cancer immunotherapy, but, only a limited number of phase III clinical trials have demonstrated clinical efficacy. The crux of this issue is the inability of tumor vaccines to effectively harmonize the tumor microenvironment with its intricate interplay. One factor that can hinder the effectiveness of vaccines is the natural immunosuppressive element present in the tumor microenvironment. This element can lead to low rates of T-cell response specific to antigens and the development of acquired resistance. Conversely, anticancer vaccines alter the tumor microenvironment in conflicting manners, inducing both immune activation and immunological evasion. Hence, comprehending the correlation between tumor vaccines and the tumor microenvironment would establish a foundation for forthcoming tumor treatment.

**Objective:**

Our review explores the realm of research pertaining to tumor vaccinations and the tumor microenvironment. Our objective is to investigate the correlation between tumor vaccines and the tumor microenvironment within this domain. We then focus our review on the dominant international paradigms in this research field and visually illustrates the historical progression and emergent patterns observed in the past.

**Methods:**

From January 1, 1999 to February 7, 2023, 1420 articles on the interplay between tumor vaccines and the tumor microenvironment were published, according to The Clarivate Web of Science (WOS) database used in our review. A bibliometric review was designed for this collection and consisted of an evaluation. The evaluation encompassed various discernible attributes, including the year of publication, the journals in which the articles were published, the authors involved, the affiliated institutions, the geographical locations of the institutions, the references cited, and the keywords employed.

**Results:**

Between the years 1999 and 2022, publications saw a significant increase, from 3 to 265 annually. With 72 papers published, Frontiers in Immunology had the most manuscripts published. The Cancer Research publication garnered the highest number of citations, amounting to 2874 citations. The United States exerts significant dominance in the subject, with the National Cancer Institute being recognized as a prominent institution in terms of both productivity and influence. Furthermore, Elizabeth M. Jaffee was recognized as the field’s most prolific and influential author with 24 publications and 1,756 citations. The co-occurrence cluster analysis was conducted on the top 197 keywords, resulting in the identification of five distinct clusters. The most recent high-frequency keywords, namely immune therapy, dendritic cell, tumor microenvironment, cancer, and vaccine, signify the emerging frontiers in the interaction between tumor vaccines and the tumor microenvironment.

**Conclusion:**

Our review uncovers insights into contemporary trends, global patterns of collaboration, fundamental knowledge, research areas of high interest, and emerging frontiers in the field of TME-targeted vaccines.

## 1 Introduction

The fundamental principle underlying cancer immunotherapy involves harnessing the inherent capabilities of the patient’s immune system to modulate the process of tumor regression. In recent years, there have been notable breakthroughs in cancer antigen vaccination research. However, it is noteworthy that only a limited number of phase III clinical studies have successfully demonstrated clinical benefit. One contributing factor to this limited success is the impediment posed by the tumor microenvironment (1). T cells are the core element of the immune response to malignancies. The main objective of therapeutic cancer vaccines is to facilitate the regression of tumors by stimulating the production of antigen-specific T cells within the body (2). Nevertheless, there exist suppressive mechanisms within TME that have the potential to restrict the functionality of T cells (3). Simultaneously, anticancer vaccines exert a dual effect on the tumor microenvironment, inducing immune stimulation as well as immune escape. The presence of inherent immunosuppressive elements within the tumor microenvironment, as well as the development of acquired resistance resulting from vaccination, both play a role in the phenomenon of vaccine resistance (1). Hence, it is imperative to enhance our comprehension of the immune milieu surrounding tumors to enhance the efficacy of personalized anti-cancer vaccines. In the past twenty years, scholarly articles have documented multiple associations between the tumor microenvironment and tumor vaccines, including certain elements within the TME facilitate the efficacy of tumor vaccines in inducing tumor cell death. Conversely, other components within the TME act as protective measures for tumor cells, offering mechanical reinforcement or releasing various cytokines to evade therapeutic interventions (4–7). Furthermore, subsequent to vaccination, certain immune components are enlisted and stimulated, while others are excluded and suppressed, resulting in an alteration of the TME configuration. The outcome of this alteration results in either immune stimulation or evasion (8, 9).

Bibliometric review is a robust and quantitative research tool employed to examine scholarly publications. Its primary objective is to provide a comprehensive overview of the advancements made within a specific research theme. Additionally, it aims to identify prominent areas of interest or emerging trends, as well as evaluate the contributions made by authors, journals, institutes, or countries through the use of quantitative statistical measures (10). VOSviewer is a software tool that facilitates the visualization of co-occurrence patterns among keywords and researchers through the creation of visual maps. The software can be accessed at http://www.vosviewer.com (11).

Despite the existence of several reviews pertaining to vaccines that target the TME with varying focuses, there remains a notable absence of a comprehensive and visually represented analysis regarding the progression and patterns of such vaccines (12–15).

By employing bibliometric review, we initially assessed the present state of research domains pertaining to vaccines that specifically target the TME, while also investigating the prevailing patterns and developments within this area. Our objective was to ascertain the prevailing hotspots in this region in order to provide insights into potential areas for future research. In addition, our review conducted in-depth analyses on significant subtopics identified through bibliometric characterization. This research endeavor would provide valuable assistance to both novice researchers and experts in the field by facilitating the identification of a comprehensive range of research topics, including the discovery of novel areas of investigation, and aiding in the strategic planning of research endeavors pertaining to vaccines targeting TME.

## 2 Methods and materials

### 2.1 Retrieval strategy and data collection

**Figure 1:**
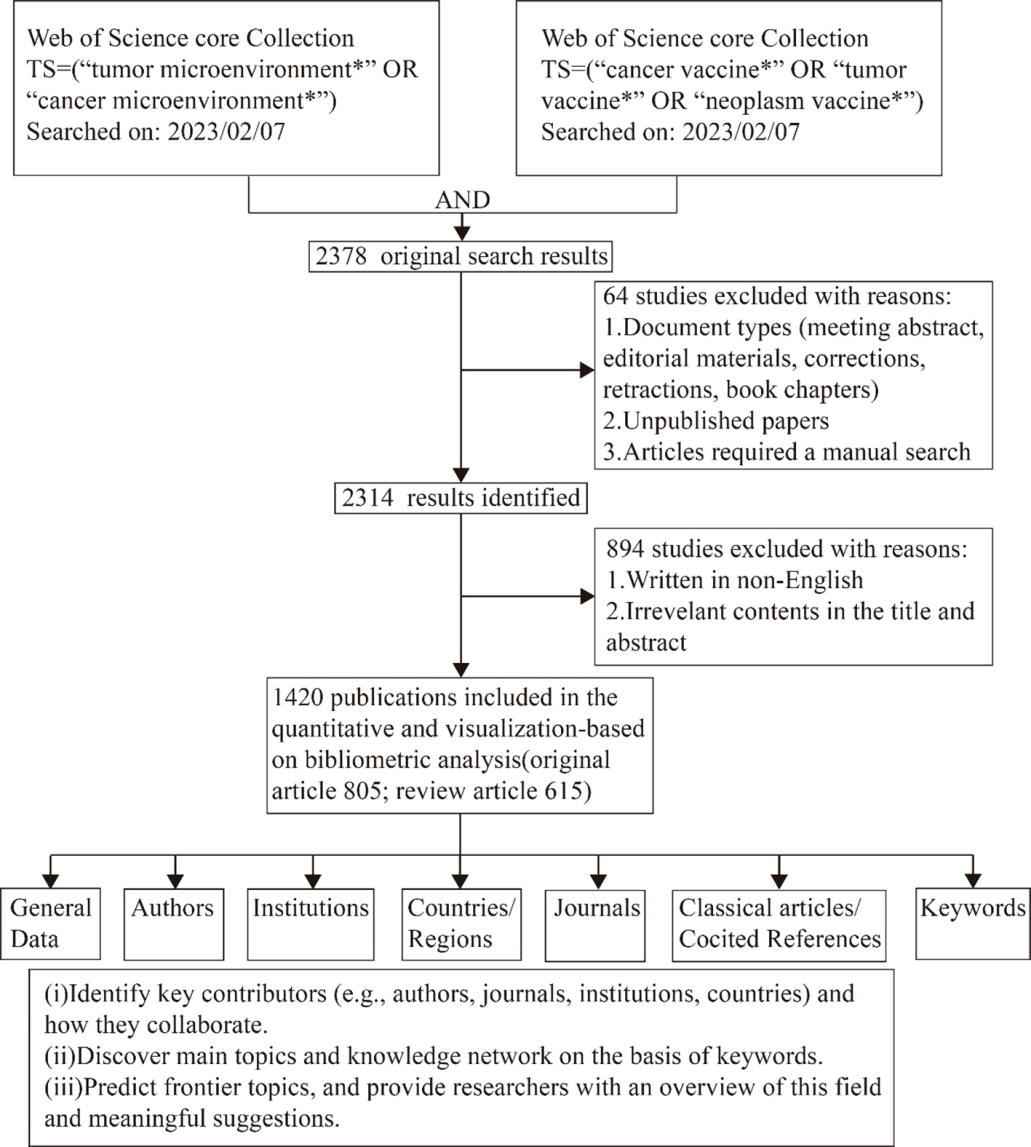
Diagram illustrating the process of data filtering and bibliometric analysis.

For this bibliometric analysis, the researchers collected publication data on a specific date (February 7, 2023). The data was obtained by downloading “Plain text” files from the Web of Science Core Collection (WoSCC). The methodology for data collection and retrieval was illustrated in **Figure 1**. The publications that were obtained needed to meet the following criteria:

(1) The search query string utilized were TS = (“tumor microenvironment*” OR “cancer microenvironment*”) and TS = (“tumor vaccine*” OR “cancer vaccine*” OR “neoplasm vaccine*”), to ascertain papers pertaining to vaccinations specifically designed for targeting TME study. The publication period considered spanned from 1999 to 2023. The data collected encompassed publication details, author information, country affiliations, institutional affiliations, journal sources, keywords, and citation counts. **Figure 1** illustrates the strategy employed for the collection and retrieval of data. The authors of this study are Ying Liu and Sixin Li. Discrepancies were handled through deliberations with the other two writers (Lu Chen and Lin Lin) until a consensus was achieved.

### 2.2 Data analysis and network mapping

The utilization of bibliometric review can contribute significantly to the monitoring of the progress and trends observed in impactful scholarly publications. In recent times, there has been a significant adoption of bibliometric visualization software for the purpose of extracting and analyzing publication data, as well as generating knowledge maps (16). The complete records and properly referenced sources of all the documents in text format were acquired and compiled from WoSCC. Bibliometric parameters such as title, keywords, authors, institutions, countries or regions, journal, publication year, total citations (TC), citations per publication (CPP), and cited references were extracted and exported to various software tools and platforms. These include CiteSpace (version 6.1.R6), VOSviewer (version 1.6.18) developed by Leiden University in the Netherlands, Microsoft Excel 2019 developed by Microsoft Corporation in the USA, two online platforms (https://flourish.studio/examples/ and https://bibliometric.com/), as well as the R Bibliometrix Package. The purpose of using these tools and platforms was to identify the largest contributors, including prolific authors, institutions, and countries. CiteSpace is an analysis tool for citation visualization in scientific literature, enabling understanding and tracking knowledge generation through visualization (17). Bibliometrix facilitates a suggested methodology for doing bibliometric analyses and may be easily enhanced and combined with other statistical R-packages (18). These data were subsequently utilized to generate visual representations of network maps. The analysis of co-authorship unveils discernible patterns of collaboration among authors, institutions, and countries (19). Co-occurrence analysis is a technique that investigates the frequency at which numerous terms occur together within a single article. It allows for the determination of the closeness between these terms, therefore offering insights on common subjects and developing patterns within the specific field. Cocitation analysis facilitates researchers in the identification and assessment of the knowledge foundation within a particular academic field (20). The purpose of our review was to conduct a co-word analysis in order to investigate the research hotspots related to vaccines that target TME. In the visualization maps of VOSviewer and CiteSpace, each node is depicted as a circular shape accompanied by a corresponding label. The co-occurrence analysis reveals that circles of larger size correspond to higher frequencies. The hue of each circular entity is contingent upon the cluster to which it is assigned. The dimensions of the links connecting nodes are indicative of the intensity and significance of the association and pertinence between the respective nodes. Node size positively correlates with the number of publications, whilst line thickness indicates the level of collaboration between the two nodes.

### 2.3 Ethics in research

The bibliographic information was retrieved and downloaded from WoSSC. The data in question were readily accessible to the public. The acquisition of this data did not entail any form of engagement with human participants or animals. Consequently, the utilization of this data did not give rise to any ethical concerns. There was no requirement for approval from an Ethics Committee.

## 3 Results

### 3.1 Annual global publication outputs on vaccines targeting the TME

**Figure 2:**
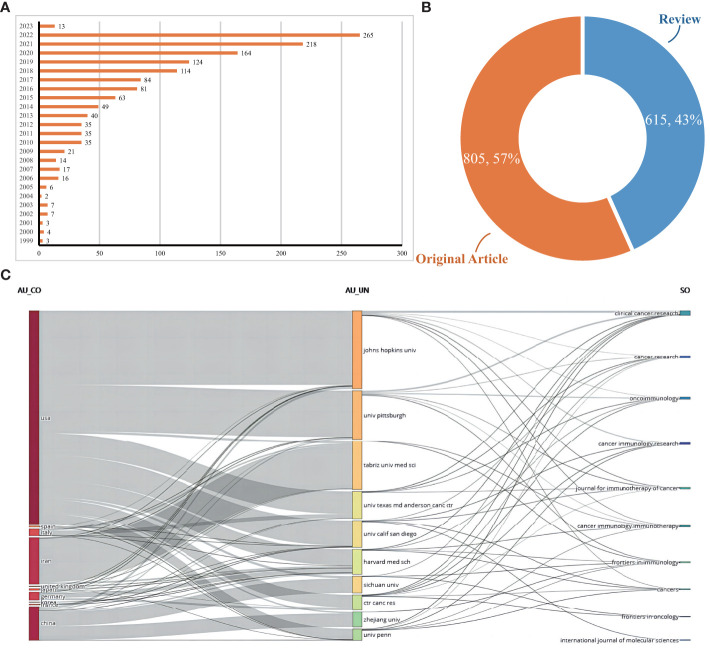
**(A)** Documents by years. **(B)** Distribution of publications type. **(C)** The relationship between the countries, institutions, and journals resulted in the publication of articles based on an alluvial flow map derived from R for vaccines targeting the TME.

A comprehensive collection of 1420 scholarly articles pertaining to vaccines specifically designed for targeting the TME was obtained from WoSCC database, spanning the time period from 1999 to 2023. **Figure 2A** displays the annual publication output pertaining to vaccines specifically designed to target TME. The field of research pertaining to vaccines targeting TME has experienced a significant surge in interest over the course of the last twenty years. The number of annual global publications experienced a significant growth, rising from a mere 3 in 1999 to a substantial 265 in 2022. The quantity of annual publications centered on vaccinations targeting TME was less than 10 between 1999 and 2005. However, between 2006 and 2012, there was a steady rise in the quantity of publications, with the output rising steadily from 16 to 35. Notably, between the years 2013 and 2019, the data indicates a significant and rapid increase in outputs, with the numbers escalating from 40 to 124. Between the years 2020 and 2022, the output surpassed a threshold of 150 and reached its highest point at 265 in the year 2022. The publications were chosen for further analysis, with 57% being original works (n = 805) and the remaining being reviews (n = 615; **Figure 2B**).

**Figure 2C** illustrates the interconnectedness among countries, institutions, and journals in the context of vaccines targeting the TME, as depicted by Three-field plots or alluvial flow maps. The plot was constructed with a grouping of ten items. The United States encompassed or established affiliations with seven specific institutions, namely Johns Hopkins University(JHU), University of Pittsburgh, University Texas MD Anderson Cancer Center, University of California San Diego(UC San Diego), Harvard Medical School(HMX), Center for Cancer Research(CCR), and University of Pennsylvania. Iran is associated with or affiliated with a specific institution, namely Tabriz University of Medical Sciences. JHU connected with seven targeted journals (Clinical cancer research, Cancer research, Oncoimmunology, Cancer immunology research, Journal for Immunotherapy of cancer(JITC), Cancer immunology immunotherapy(CII), and Cancers); University of Pittsburgh connected with six targeted journals (Clinical cancer research, Cancer research, Oncoimmunology, Journal for Immunotherapy of Cancer, Cancer Immunology Immunotherapy and Frontiers in Immunology); Tabriz University of Medical Sciences connected with two targeted journals (Cancer Immunology Immunotherapy and Frontiers in Inmmunology); University Texas MD Anderson Cancer Center connected with four targeted journals (Clinical cancer research, Cancer research, Oncoimmunology, and Journal for Immunotherapy of Cancer). It is noteworthy that a significant proportion of collaborations among institutions in the United States and Iran primarily occurred within their own respective institutional frameworks.

### 3.2 Distribution of the top cited journals and articles

**Figure 3:**
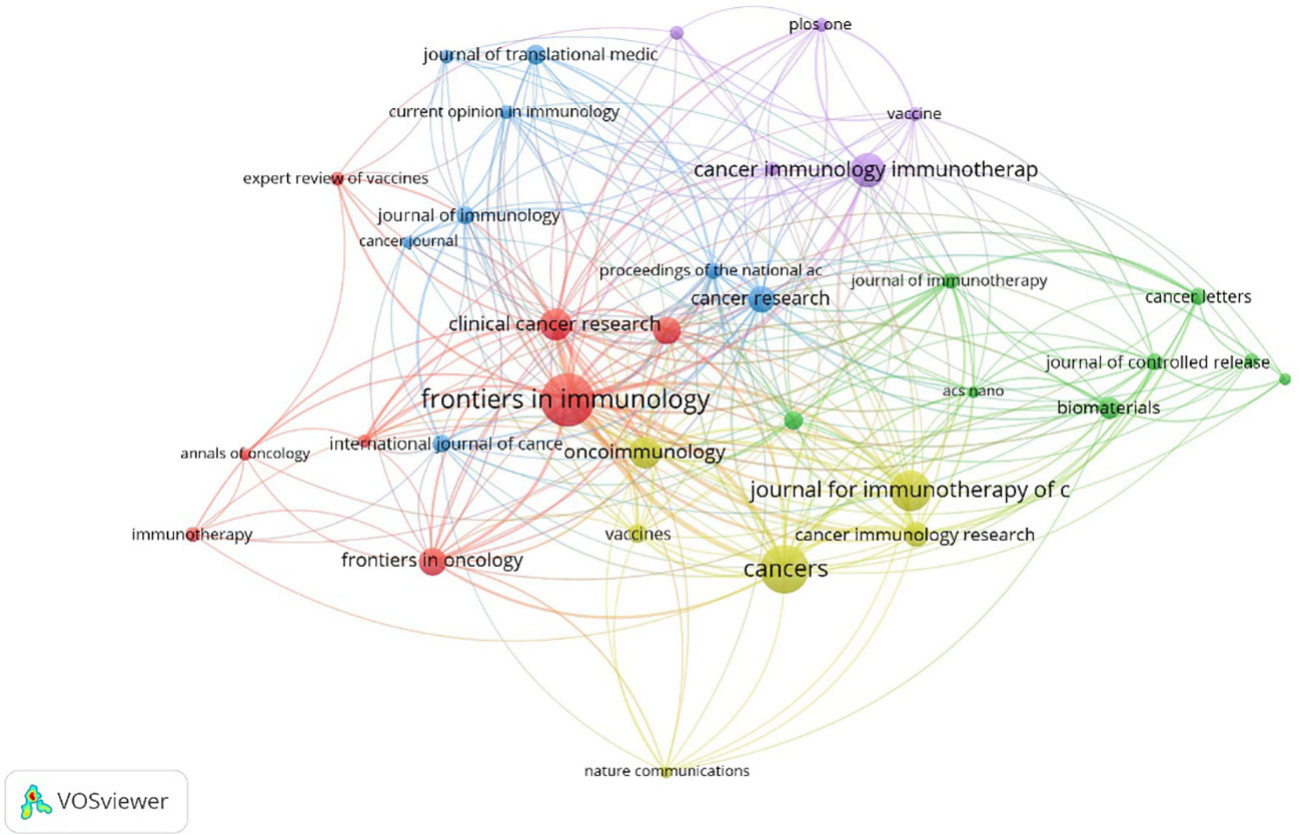
Collaboration among journals. The size of a node is indicative of the quantity of articles it represents. The width of connections serves as an indicator of the strength of collaboration.

The articles pertaining to vaccines targeting the TME were published in a total of 422 academic journals. **Table 1** presents a compilation of the ten most prominent academic journals that have published a substantial number of articles pertaining to vaccines targeting the TME. These journals collectively account for 27.5% (391 out of 1,420) of the total publications in this field. According to **Table 1** and **Figure 3**, the journal “Frontiers in Immunology” exhibited the highest degree of productivity, with a total of 72 publications. Subsequently, the journal “Cancers” demonstrated a commendable level of productivity with 62 publications, while the “Journal for Immunotherapy of Cancer” showcased a respectable level of productivity with 48 publications. Of the top ten cited articles (**Table 2**), the journal “Cancer Research” received the highest number of citations, totaling 2,874. This count was derived from 26 articles, resulting in a median citation count of 110.5385. **Table 2** presents a compilation of the ten most frequently referenced scholarly articles pertaining to vaccines designed to target the TME. The range of citations for the top 10 most cited articles varied from 426 to 1,556. The article titled “Regulatory T Cells, Tumor Immunity and Immunotherapy,” which was published in the esteemed journal Nature Reviews Immunology in 2006, has garnered the most citations, with a total of 1,556 citations. **Table 3** demonstrates that 90% (9/10) of the top ten co-cited journals originated from the United States of America (USA), whereas 10% (1/10) were from the United Kingdom (UK). According to the Journal Citation Reports (JCR), these journals are classified as either Q1 or Q2. Among these, nine journals are positioned in Q1 across several fields.

**Table 1:** Top 10 prolific journals for tumor microenvironment and tumor vaccine.

Rank	Journal	Documents	TC	CPP	IF (2022)	JCR (2022)
1	Frontiers in Immunology	72	1,538	21.3611	8.787	Q1
2	Cancers	62	781	12.5968	6.575	Q1
3	Journal for Immunotherapy of Cancer	48	475	9.8958	12.485	Q1
4	Cancer Immunology Immunotherapy	38	1,018	26.7895	6.63	Q1
5a	Clinical Cancer Research	33	2,229	67.5455	13.801	Q1
5b	Oncoimmunology	33	855	25.9091	7.723	Q1
7a	Frontiers in Oncology	28	647	23.1071	5.738	Q2
7b	International Journal of Molecular Sciences	28	330	11.7857	6.208	Q1
9	Cancer Research	26	2,874	110.5385	13.312	Q1
10	Cancer Immunology Research	23	1,374	59.7391	12.02	Q1

**Table 2:** The top 10 highest cited articles.

Rank	Title	Type	First Author	Journal	Year	Citations	Ref
1	Regulatory T Cells, Tumor Immunity and Immunotherapy	Review	Zou, Wp	Nature Reviews Immunology	2006	1,556	(21)
2	Pd-1 and Ctla-4 Combination Blockade Expands Infiltrating T Cells and Reduces Regulatory T and Myeloid Cells Within B16 Melanoma Tumors	Article	Curran, Michael A.	Proceedings of the National Academy of Sciences of the United States of America	2010	1,269	(22)
3	Mutant Mhc Class Ii Epitopes Drive Therapeutic Immune Responses to Cancer	Article	Kreiter, Sebastian	Nature	2015	738	(23)
4	Neoantigen Vaccine Generates Intratumoral T Cell Responses in Phase Ib Glioblastoma Trial	Article	Keskin, Derin B.	Nature	2019	659	(24)
5	Vascular Normalizing Doses of Antiangiogenic Treatment Reprogram the Immunosuppressive Tumor Microenvironment and Enhance Immunotherapy	Article	Huang, Yuhui	Proceedings of the National Academy of Sciences of the United States of America	2012	623	(25)
6	Dendritic Cells in Cancer Immunology and Immunotherapy	Review	Wculek, Stefanie K.	Nature Reviews Immunology	2020	536	(26)
7	Vaccines for Established Cancer: Overcoming the Challenges Posed by Immune Evasion	Review	Van Der Burg, Sjoerd H.	Nature Reviews Cancer	2016	461	(27)
8	Pd-1-Expressing Tumor-Infiltrating T Cells Are A Favorable Prognostic Biomarker In Hpv-Associated Head And Neck Cancer	Article	Badoual, Cecile	Cancer Research	2013	452	(28)
9	Targeting Tumor-Associated Macrophages as a Novel Strategy against Breast Cancer	Article	Luo, Yunping	Journal of Clinical Investigation	2006	449	(29)
10	Role of Local Radiation Therapy in Cancer Immunotherapy	Review	Demaria, Sandra	Jama Oncology	2015	426	(30)

**Table 3:** Top ten co-cited journals related to the research for tumor microenvironment and tumor vaccine.

Rank	Journal	Co-citation	IF(2022)	JCR(2022)	Country
1	Cancer Research	5,041	13.312	Q1	USA
2	Clinical Cancer Research	4,733	13.801	Q1	USA
3	Journal of Immunology	4,110	5.430	Q2	USA
4	Journal of Clinical Oncology	3,393	50.739	Q1	USA
5	Nature	2,851	69.504	Q1	UK
6	New England Journal of Medicine	2,616	176.082	Q1	USA
7	Proceedings of the National Academy of Sciences of the United States of America	2,473	12.779	Q1	USA
8	Journal of Experimental Medicine	2,375	17.579	Q1	USA
9	Blood	2,350	25.669	Q1	USA
10	Science	2,269	63.832	Q1	USA

### 3.3 Top contributing countries and institutions

**Figure 4:**
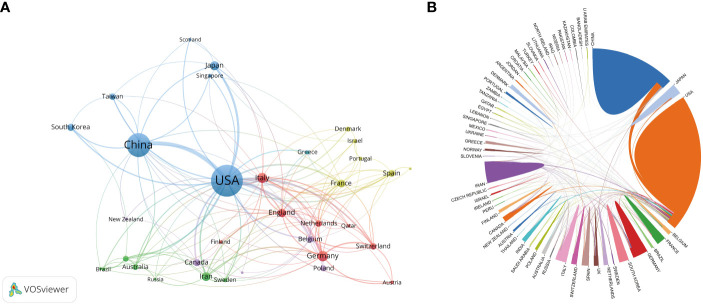
**(A)** The network of co-authorship among countries. **(B)**The cross-country/region collaborations visualization map. The magnitude of the boundary line separating nations is indicative of the extent of collaborative interactions.

The subject of vaccines targeting TME has garnered significant interest globally. A comprehensive analysis reveals that a total of 66 countries/regions have actively contributed to the study of vaccines targeting the TME, as depicted in **Figure 4A**. **Figure 4A** displays the network of co-authorship among countries/regions. The co-authorship network was partitioned into six clusters, each represented by a distinct color, encompassing 34 out of 66 countries/regions. The predominant cluster, denoted by the color blue, encompasses a total of seven countries, with a focal point on the US, China, and Japan. The US exhibited the highest count of collaborating entities (n = 30), with England (n = 21), Italy (n = 20), China (n = 18), and Germany (n = 16) following suit. The results of this study indicate that the US and China were the top two countries in terms of the number of articles, citations, and total link strength. The US had 663 articles, 34,202 citations, and a total link strength of 264, while China had 377 articles, 7,593 citations, and a total link strength of 102. These findings demonstrate that both countries had a significant presence in terms of publications, citations, and links compared to other countries, as shown in **Table 4** and **Figure 4B**.

**Table 4:** The top 10 highly prolific countries/regions for tumor microenvironment and tumor vaccine.

	Documents	TC	CPP	TLS	Links
USA	663	34,202	51.6	264	30
China	377	7,593	20.1	102	18
Germany	72	2,436	33.8	68	16
Italy	69	1,402	20.3	51	20
Japan	53	3,510	66.2	32	8
Iran	50	815	16.3	43	11
England	49	1,658	33.8	56	21
France	40	1,334	33.4	45	15
Canada	34	1,367	40.2	25	10
South Korea	34	599	17.6	14	4

TLS, Total link strength.

### 3.4 Institutional co-authorship and distribution

**Figure 5:**
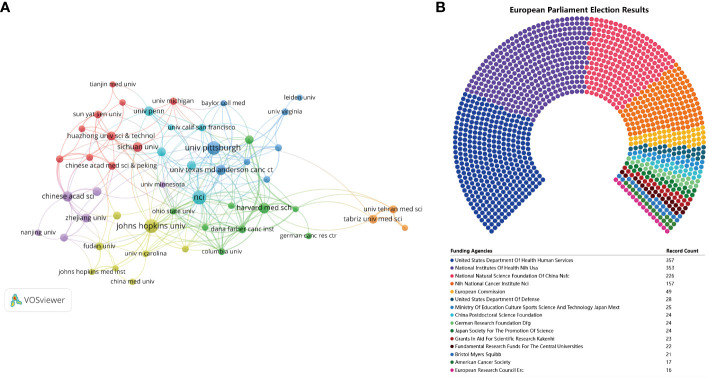
**(A)** The co-authorship network of institutions. **(B)**The top 15 most active funding agencies in microenvironment-related tumor vaccine.

A comprehensive number of 1,647 institutions made contributions towards the development of vaccines specifically designed to target the TME in biomedical research. **Table 5** displays the top 9 institutions and highly cited institutions. The National Cancer Institute demonstrated the highest level of productivity with 52 publications and 2,281 citations. Following closely behind were Johns Hopkins University with 50 publications and 3,134 citations, and the University of Pittsburgh with 47 publications and 2,548 citations. When the criterion for the minimal quantity of publications disseminated by institutions was established as 10, it was found that 50 institutions satisfied this requirement. The software tool VOSviewer was utilized to conduct a co-authorship analysis of the 50 institutions that demonstrated high productivity. **Figure 5A** displays the network of co-authorship among institutions. The co-authorship network, comprising 50 institutions, was partitioned into 7 clusters, each denoted by distinct colors. The Dana-Farber Cancer Institute exhibited the highest total link strength (TLS) with a value of 38, while The University of Texas MD Anderson Cancer Center, National Cancer Institute, and Harvard Medical School followed closely with TLS values of 32, 32, and 30, respectively. **Figure 5B** displays the top 15 most active funding agencies in microenvironment-related tumor vaccine. United States Department Of Health Human Services is the most funded organization, followed closely by National Institutes of Health (NIH), USA.

**Table 5:** The top 9 most productive institutions (left) and highly referenced institutions (right) for tumor microenvironment and tumor vaccine.

Rank	Institution	Documents	TC	CPP	TLS	Institution	Documents	TC	CPP	TLS
1	National Cancer Institute	52	2,281	43.9	354	Johns Hopkins University	50	3,134	62.7	574
2	Johns Hopkins University	50	3,134	62.7	574	University of Pittsburgh	47	2,548	54.2	347
3	University of Pittsburgh	47	2,548	54.2	347	Harvard university	19	2,345	123.4	134
4	Harvard Medical School	30	1,632	54.4	388	National Cancer Institute	52	2,281	43.9	354
5	University of Texas MD Anderson Cancer Center	28	994	35.5	236	Massachusetts General Hospital	10	2,240	224.0	306
6	Chinese Academy of Sciences	27	787	29.1	234	University of Michigan	14	2,071	147.9	148
7	Sichuan University	25	500	20.0	196	Memorial Sloan Kettering Cancer Center	13	2,031	156.2	262
8	University of Pennsylvania	24	1,339	55.8	223	Dana-Farber Cancer institute	17	1,928	113.4	243
9	Jilin University	21	359	17.1	132	University of Chicago	20	1,694	84.7	173

### 3.5 Distribution and co-authorship of authors

**Figure 6:**
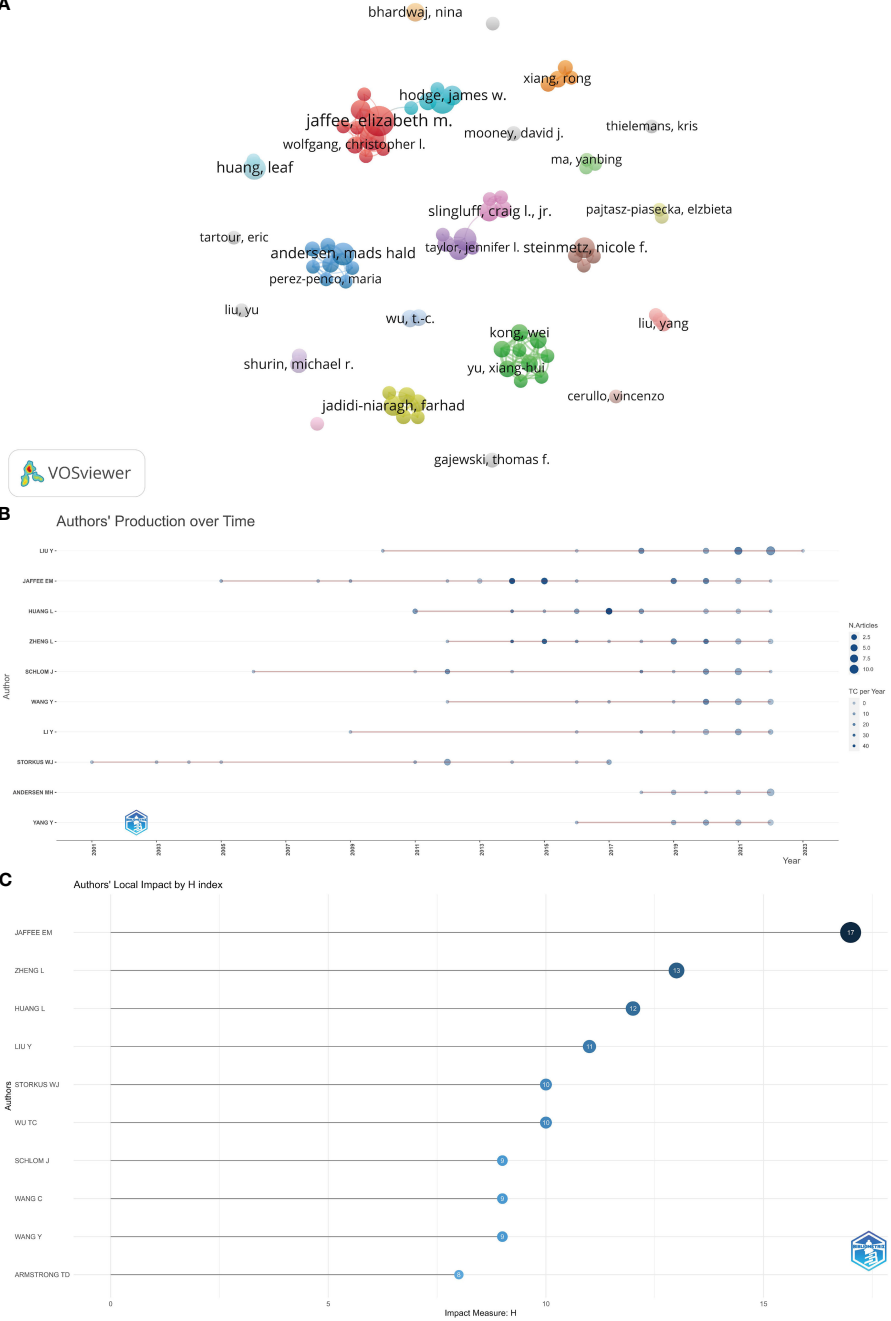
**(A)** The co-authorship network of authors. **(B)** The publication schedule for the ten most prolific authors according to R. **(C)** Author local impact based on H-index in R.

The number of authors who contributed to the 1,420 articles that were retrieved was 7,587. The authors who have shown the highest degree of production are presented in **Table 6**. According to the available data, Jaffee, Elizabeth M. has shown the highest degree of productivity, with a total of 24 publications and 1,756 citations. Following closely behind is Schlom, Jeffrey, with 16 publications and 552 citations, while Zheng, Lei has contributed 15 publications and received 1,106 citations. In the present investigation, the utilization of VOSviewer was employed to conduct an analysis on co-authorship. The minimum threshold for the quantity of articles authored by the individual is established at 5. Out of the total population of 7,587 authors, a subset of 77 authors satisfies the specified criteria. The authors’ co-authorship network was depicted in **Figure 6A**. The co-authorship network, comprising a total of 77 authors, was partitioned into 24 distinct clusters, each of which was visually represented by a unique color. The largest cluster, comprised of nine authors, was centered around Jaffee, Elizabeth M., Zheng, Lei, and Armstrong, Todd D. Elizabeth M. Jaffee had the highest number of collaborating partners, with a total of nine (n = 9).

**Table 6:** The 12 most productive authors.

Rank	Author	Documents	TC	CPP	TLS	Country/Region	Institution
1	Jaffee, Elizabeth M.	24	1,756	73.2	156	USA	Johns Hopkins University
2	Schlom, Jeffrey	16	552	34.5	58	USA	National Cancer Institute
3	Zheng, Lei	15	1,106	73.7	126	USA	Johns Hopkins University
4a	Huang, Leaf	13	1,010	77.7	7	USA	University of North Carolina System
4b	Andersen, Mads Hald	13	89	6.8	92	Denmark	University of Copenhagen
4c	Storkus, Walter J.	13	503	38.7	58	USA	University of Pittsburgh
7	Steinmetz, Nicole F.	11	200	18.2	24	USA	University of California San Diego
8a	Armstrong, Todd D.	10	337	33.7	67	USA	Johns Hopkins University
8b	Hodge, James W.	10	289	28.9	24	USA	National Cancer Institute
8c	Jadidi-Niaragh, Farhad	10	399	39.9	79	Iran	Tabriz University of Medical Sciences
8d	Okada, Hideho	10	347	34.7	67	USA	University of California San Francisco
8e	Slingluff, Craig L., Jr.	10	293	29.3	69	USA	University of Virginia

Furthermore, in accordance with the publishing chronology for the ten most prolific authors depicted in **Figure 6B**, Liu Y, Jaffee EM, Huang L, Zheng L, School J, Wang Y, LI Y, Stories WJ, Andersen MH, and Yang Y possessed the most extensive duration in terms of publishing on tumor microenvironment and tumor vaccine. Jaffee EM had the highest number of impact measures (H-index, 17), followed by Zheng L (H-index, 13), Huang L (H-index, 12), and Liu Y (H-index, 11) (**Figure 6C**).

### 3.6 Co-citation references analysis

**Figure 7:**
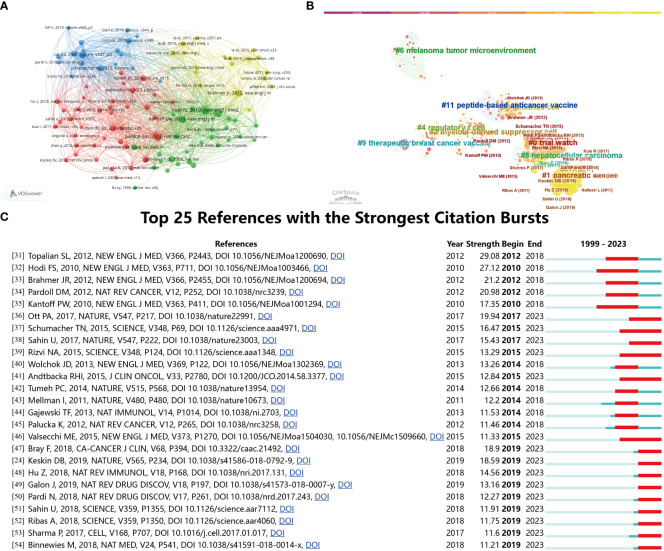
**(A)** The co-citation network visualization map of references on tumor microenvironment and tumor vaccine. **(B)** Reference co-citation network clustered by CiteSpace. **(C)** The top 25 references with the strongest citation bursts on tumor microenvironment in tumor vaccine field.

The 1,420 retrieved publications cited 37,907 references. **Figure 7A** displays network map the co-citation network visualization map of references. The paper titled “Vaccines targeting the TME” published in NEW ENGL J MED in 2012, was the most cited reference. In order to investigate the evolutionary trajectory of vaccines targeting the TME, a co-citation study was performed utilizing the software tool CiteSpace. According to the data presented in **Figure 7B**, the network exhibits publications that rank top 10 per cent of references. The articles that have been published from 2019 until the present have mostly focused on pancreatic cancer. The predominant subjects of debate in papers published between 2014 and 2018 were “hepatocellular carcinoma” and “trial watch,” indicating their significance throughout that period. Furthermore, a citation explosion methodology was employed to ascertain the noteworthy references that have made substantial contributions to the existing knowledge in this particular sector. **Figure 7C** displays the top 25 references with the most significant citation bursts (24, 31–54). The upward trend in citations within this domain started in 2010, and several co-citation references continued to be extensively cited in subsequent years, suggesting that the investigation of the tumor microenvironment in the field of tumor vaccines has remained a prominent area of study.

### 3.7 The co-occurrence analysis of keywords

**Figure 8:**
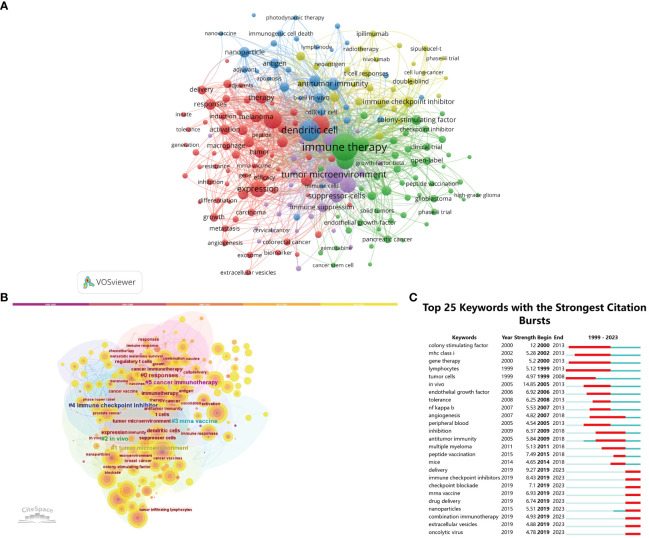
**(A)** The co-occurrence networks of keywords are visualized by VOSviewer. The terms with high frequency were shown as large nodes, while nodes of the same color denoted closer associations. **(B)** Keywords clusters named by the CiteSpace LLR algorithm from 1999 to 2023. **(C)** The top 25 keywords with the strongest citation bursts on tumor microenvironment in tumor vaccine field.

The primary topics covered in scholarly papers are denoted by keywords, thereby making high-frequency keywords particularly suitable for inclusion in co-occurrence analysis. Our bibliometric review involved the extraction and clustering of the top 100 keywords, as seen in **Table 7**, through the use of VOSviewer and CiteSpace. The network map visualization in **Figure 8A** illustrates the arrangement of the top 197 keywords into five separate clusters, showcasing their co-occurrence. Thesaurus (**Supplementary Material**) was employed to eliminate duplicate keywords, such as antibodies were replaced by antibody.

**Table 7:** Clusters of the top 100 keywords.

Cluster	Keywords	Counts	Rank	Cluster	Keywords	Counts	Rank
1	cancer	274	4	2	checkpoint blockade	43	59
1	vaccine	268	5	2	oncolytic virus	43	60
1	expression	203	8	2	monoclonal antibody	41	66
1	therapy	117	13	2	endothelial growth-factor	40	69
1	breast neoplasms	104	14	2	natural killer cell	38	74
1	melanoma	100	15	2	checkpoint inhibitor	37	77
1	tumor	99	17	2	phase-ii trial	37	78
1	responses	98	18	2	lung cancer	36	82
1	vaccination	94	21	2	immune checkpoint	33	90
1	activation	90	22	2	antitumor-activity	32	93
1	cells	89	23	2	targeted therapy	32	94
1	microenvironment	86	24	2	adoptive immunotherapy	30	99
1	immunity	84	27	2	gene therapy	30	100
1	delivery	65	35	3	dendritic cell	374	2
1	growth	63	36	3	antitumor immunity	128	12
1	macrophage	60	38	3	cancer immunotherapy	96	19
1	blockade	59	39	3	nanoparticle	95	20
1	survival	59	40	3	*in-vivo*	86	25
1	lymphocytes	54	42	3	antigen	81	28
1	mechanism	47	50	3	cancer-immunotherapy	74	30
1	carcinoma	46	51	3	cd8(+) t cell	51	47
1	colorectal cancer	44	55	3	tumor-associated macrophage	41	67
1	induction	43	57	3	*in-vitro*	40	70
1	receptor	43	58	3	adjuvant	38	75
1	efficacy	42	61	3	immunogenic cell death	37	79
1	Metastasis26	41	64	3	drug delivery	36	83
1	tumor antigen	41	65	4	immune checkpoint inhibitor	100	16
1	hepatocellular carcinoma	39	71	4	chemotherapy	74	31
1	immunogenicity	39	72	4	metastatic melanoma	72	32
1	inhibition	37	76	4	combination	69	34
1	angiogenesis	36	80	4	prostate cancer	54	43
1	exosome	36	81	4	t-cell responses	53	45
1	inflammation	35	84	4	pd-1	51	48
1	differentiation	33	88	4	double-blind	46	53
1	progression	33	89	4	antibody	42	62
1	identification	31	97	4	ipilimumab	41	68
1	tumor vaccine	30	98	4	pd-l1	39	73
2	immune therapy	596	1	4	trial	34	85
2	t cell	253	6	4	neoantigen	33	91
2	cancer vaccine	166	9	4	combination therapy	32	95
2	colony-stimulating factor	85	26	4	safety	32	96
2	tumor-infiltrating lymphocytes	78	29	5	tumor microenvironment	278	3
2	phase-i	63	37	5	regulatory t cells	205	7
2	open-label	59	41	5	suppressor-cells	133	10
2	pancreatic cancer	53	44	5	immune-response	130	11
2	clinical trial	51	46	5	immune suppression	72	33
2	pd-1 blockade	50	49	5	tgf beta	42	63
2	glioblastoma	46	52	5	dna vaccine	34	86
2	phase-i trial	45	54	5	peptide vaccine	34	87
2	phase-ii	44	56	5	cytokine	33	92

The central nodes in the visualization network map consist of the following key terms: immune therapy (596), dendritic cell (374), tumor microenvironment (278), cancer (274), and vaccine (268). VOSviewer automatically organized keywords exhibiting similarities into five distinct clusters. Cluster 1, depicted in red, is associated with the topics of cancer and vaccines. Cluster 2, represented by the color green, is associated with the field of immune therapy. Cluster 3, denoted by the color yellow, is associated with the concepts of dendritic cells and immune checkpoint inhibitors. Cluster 4, depicted in blue, is associated with the topic of immune checkpoint inhibitors. Lastly, Cluster 5, represented by the color purple, is associated with the concept of the tumor microenvironment. As displayed in **Figures 8A, B**, in order to examine the temporal patterns of evolution, the keywords obtained from the publications were subjected to coloration in VOSviewer and CiteSpace, utilizing their average appearance year (AAY) as a basis for coding. The keywords that have emerged recently include immune therapy, dendritic cell, tumor microenvironment, cancer, vaccine, T cell, regulatory T cells, cancer vaccine, suppressor cells, and immune response. An further significant indicator of the advancements in research frontiers, areas of intense activity, and emerging patterns over time was the magnitude of the bursts seen in the keywords (**Figure 8C**). Notably, the citation burst time of terms such as “colony stimulating factor” (2000–2013), “delivery” (2019–2023), “immune checkpoint inhibitors” (2019–2023), “peptide vaccination” (2015–2018), “checkpoint blockade” (2019–2023), and “mrna vaccine” (2019–2023) has sustained into 2023, with ongoing signs indicating a notable surge in scholarly interest within specific fields of research.

## 4 Discussion

### 4.1 Global trends in vaccines targeting the TME research

Following an extensive period of knowledge acquisition, research on vaccines targeting TME has entered a phase of accelerated advancement. The quantity of scholarly articles pertaining to vaccines that focus on TME has exhibited a consistent upward trend over the course of the previous twenty years, as visually represented in **Figure 2A**. While there have been minor variations in certain years, the quantity of articles pertaining to vaccines targeting the TME has exhibited a consistent pattern of nearly doubling every five years. Notably, within the most recent five-year period, these articles constituted approximately 56.16% of the total articles published on this subject over the past two decades. The subject of vaccines that specifically target TME has garnered significant interest across various academic disciplines. A considerable number of authors, totaling 7,587, affiliated with 1,647 research institutions across 66 countries, have contributed to the publication of articles pertaining to vaccines targeting TME. This extensive participation indicates a significant global interest in the field of vaccines targeting TME research. As depicted in **Figures 4A**, **5A**, there was a notable degree of collaboration observed among countries and regions, with no discernible limitations imposed by geographical boundaries. The United States of America (USA) has historically been recognized as a highly productive nation and a central hub for international collaboration. Therefore, the United States of America emerged as a leading force in both scientific and academic research.

Out of the nine most productive institutions, six were linked with institutions based in the United States, while the other three were associated with schools based in China.

American institutions were the primary entities in terms of quantity when it came to conducting research on vaccines targeting TME. According to the data presented in **Figure 5A**, a significant majority of institutions that participated in the development of vaccines targeting TME were part of the cooperation network. Nonetheless, it is worth noting that there was limited collaboration between institutions within the same country. Specifically, the blue cluster was predominantly composed of American institutions, while the red cluster was predominantly dominated by Chinese institutions. According to the data presented in **Table 6**, Jaffee, Elizabeth M. emerged as the most prolific author in terms of productivity. 

### 4.2 Knowledge base, hotspots, and emerging Frontiers on vaccines targeting the TME research

Efficient revelation of the knowledge base and background pertaining to vaccines targeting TME can be achieved through the utilization of co-citation analysis on cited references. According to the data presented in **Table 2**, the top 10 cited references encompass scholarly investigations pertaining to the field of oncology. The topics of interest include immunity, immunotherapy, neoantigen vaccine, neoantigens, and recent advancements in these areas. The analysis of keyword co-occurrence has provided support for the categorization of the primary knowledge framework and areas of significant interest. According to the findings presented in **Figure 8A**, the vaccines targeting the tumor microenvironment research conducted between 1999 and 2023 can be categorized into five distinct research clusters. These clusters were identified through co-occurrence cluster analysis of the top 197 keywords associated with this field of study.

#### 4.2.1 Cancer

According to estimates, there were around 19.3 million newly diagnosed cases of cancer globally in the year 2015, leading to over 10.0 million fatalities (excluding nonmelanoma skin cancer). The incidence of female breast cancer has now exceeded that of lung cancer, making it the most frequently diagnosed cancer. According to estimates, the projected number of newly diagnosed cases of female breast cancer is roughly 2.3 million, representing approximately 11.7% of all cancer diagnoses. The subsequent leading cancer types in terms of prevalence are lung cancer, colorectal cancer, prostate cancer, and stomach cancer (55). The escalating incidence of cancer and the significant mortality rate underscore the limitations and suboptimal outcomes associated with existing cancer therapeutic strategies. Melanoma constitutes approximately 1.7% of cancer diagnoses worldwide and ranks as the fifth most prevalent cancer in the United States. Melanoma is becoming more common in wealthy, mostly fair-skinned countries. Since 1975, the number of cases in the US has risen by more than 320% (56). Consequently, there has been a shift in research emphasis towards the development and progression of novel methodologies for cancer diagnosis and therapy within the field of cancer treatment.

Tumors exhibit heterogeneity from patient to patient, from different lesions in the same patient, and at the molecular and phenotypic levels. The current study demonstrates that the application of combination immunotherapies reduces the plasticity-driven resistance of tumor cells (57). In recent years, the field of tumor treatment has witnessed notable advancements attributed to immunotherapy. Targeting Cytotoxic mast cells, tumor-associated macrophages, and tumor-associated myeloid cells can synergize immunotherapy (58–60). Current immunotherapies for tumors include checkpoint inhibitors, adoptive cell therapy, monoclonal antibodies, anticancer vaccines, and CAR-T cells (61, 62). Meanwhile, nanoparticle delivery systems are also used in the immunotherapy of tumors (63). Nanodrug delivery systems (NDDS) employ nanoparticles to encapsulate drug carriers, enabling precise targeting of the tumor site with exceptional stability and biocompatibility. This approach extends the duration of drug activity and significantly diminishes the likelihood of severe side effects (64). Nevertheless, the potent immunosuppressive properties of TME diminish the efficacy of cancer immunotherapy (65). Consequently, the examination of TME has become indispensable in the context of tumor immunotherapy.

TME consists of a diverse array of cellular and non-cellular constituents, encompassing cancer cells, stromal cells, blood vessels, nerve fibers, extracellular matrix, and related acellular components. TME functions as a habitat for cancer cells and acts as a conduit that links cancer with the entire organism. The hypoxic TME induces significant cellular stress, leading to increased heterogeneity and plasticity of tumors. This phenomenon plays a crucial role in the emergence of more invasive tumor phenotypes that are resistant to therapeutic interventions (66). Hypoxia induces the activation of vascular endothelial cells, leading to an upregulation of transcription of vascular endothelial growth factor (VEGF) and the stimulation of excessive angiogenesis. This process significantly impacts TME and the effectiveness of therapeutic interventions. It should be noted that the hypoxic tumor microenvironment has the ability to facilitate angiogenic mimicry (VM). This process entails the creation of microvascular channels consisting of tumor cells. Consequently, VM has become prominent as a novel paradigm for the development of neovascularization in highly aggressive tumors, thereby enabling the provision of blood supply to sustain tumor growth (67). Simultaneously, anticancer vaccines reshape the tumor microenvironment in contradictory ways, causing both immune stimulation and immune escape. The innate immunosuppressive component of the tumor microenvironment and acquired resistance due to vaccination can contribute to vaccine resistance (1). Therefore, clarification of the mechanism of action between the tumor microenvironment and tumor vaccines would be of great benefit to tumor therapy.

#### 4.2.2 Tumor microenvironment

In recent years, scientists have identified numerous pathways to combat tumors by promoting TME conditions and thereby increasing the precision of targeted therapies. These include Treg cells, myeloid-derived suppressor cells, Transforming growth factor-β (TGFβ) and cancer-associated fibroblasts (CAFs). Tregs exert an influence on the transcriptional programmes of crucial accessory cells within the tumor microenvironment (68). Although strategies for depleting Tregs in patients with tumors have shown some success, their overall clinical efficacy is constrained and accompanied by adverse side effects (69). Myeloid-derived suppressor cells (MDSCs) have been recognized as crucial constituents of TME and play a role in promoting immune tolerance towards tumors. Modulating the recruitment, expansion, activation, and immunosuppression of MDSCs has the potential to partially reinstate the body’s ability to mount an effective antitumor immune response (70). TGFβ not only exerts anti-tumor activity in premalignant cells by inducing apoptosis or cell cycle blockade, but also promotes tumor-promoted polarization of innate immune cells or cancer-associated fibroblasts (71). There is an increasing amount of data indicating the pleiotropic nature of TGF-β signaling as a crucial pathway in the development of a fibrotic TME. This environment consists of various components, including CAFs, extracellular matrix proteins, and remodeling enzymes (72). ATF-mediated translocation of exosomes into fibroblasts and activation of the SMAD pathway drive CAF differentiation by cancer cells (73). Enrichment of CAFs in TME accelerates malignant progression of tumors by remodeling the pre-culture tumorigenic ecological niche (74). Multiple studies have demonstrated the ability of mRNA vaccines, *in situ* anti-tumor vaccines, CCL22-based peptide vaccines, and novel bioactive nanovaccines to enhance the immune microenvironment of tumors. This transformation shifts the tumors from an immunosuppressive state to an immunostimulatory state, resulting in more effective anti-tumor effects (13, 14, 75–77). Shukla, P., et al. discovered that 3D bioprinting has the capability to accurately replicate the intricate composition of TMEs, hence creating a potential avenue for the advancement of efficient drug screening methods (78).

#### 4.2.3 Immune therapy

Tumor-infiltrating immune cells (TLSs) possess multiple immune cell types in a single tumor microenvironment, and each cell type exhibits multiple states (79). The principle of immunotherapy is to activate our own immune system to defeat cancer. Currently, it mainly includes cytokines, antibody depletion, cell transplantation therapy, oncolytic virus, cancer vaccines, and immune checkpoint inhibitors (ICIs) (80). Among them, ICI is the most widely used cancer immunotherapy. The precise detection and localization of tumor-infiltrating lymphocytes (TILs), together with their spatial arrangement and sophisticated immunological structures such tertiary lymphoid structures (TLSs), play a critical role in the correct prognostication of cancer. Recent studies have shown that anti-PD-1 therapy, by activating TPE cells, has become a key factor in improving the response rate and clinical prognosis of cancer patients undergoing immunotherapy. For example, the simultaneous targeting of NK cells and T cells through the use of anti-NKG2A and anti-TIGIT drugs, which are immune checkpoint inhibitors, has already entered clinical trials (80, 81). Colony-stimulating factor (CSF) promotes the activation of T-cell immune responses by antigen-presenting cells and the enhancement of antibody-dependent cell-mediated cytotoxicity by macrophages against tumors. Recent research findings have indicated that the combination of GM-CSF with radiotherapy, immune checkpoint inhibitors, and therapeutic vaccines for tumors is clinically more effective than the use of GM-CSF alone in immunotherapy for tumors (82–85). Du, Y., et al. created PMA-captured neoantigenic vaccinations that enhance the administration of the tumor vaccine and also increase the immunogenicity of the vaccine. The combination of PMA-NeoV and IPI-549, a molecular regulator of immunosuppression, effectively suppressed tumor development by converting suppressive macrophages into an active state and stimulating T cells to generate a strong tumor immune milieu (86). Moreover, Badrinath, Set al. developed a vaccine that inhibits the hydrolytic shedding of MICA/B proteins from tumor cells, which induces tumor immunity via T and NK cells while avoiding the induction of antibodies that may block NKG2D receptor binding. It is effective in metastatic and drug-resistant tumors and has potential for clinical application (87).

#### 4.2.4 Dendritic cell

Cross-presentation of tumor-associated antigens by antigen-presenting dendritic cells (DCs) to cytotoxic CD8+ T cells (CTLs) (88). DC-based vaccines have been authorized as a means to effectively stimulate targeted immune responses against tumor cells. However, the limited effectiveness of DC vaccines is primarily attributed to their suboptimal design and the presence of an immunosuppressive tumor microenvironment, which hampers their ability to combat tumors (89). Toll-like receptors (TLRs) are a group of pattern-recognition receptors that are essential for identifying pathogen-associated chemical patterns and activating immune cells to initiate an immune response (90). TLR agonists have been employed as immunoadjuvants in order to enhance the effectiveness of cancer immunotherapies (90). TLRs are situated within the plasma membranes of cells as well as intracellular endosomes. They possess the ability to recognize various pathogen-associated molecular patterns originating from bacteria, viruses, and fungi (91). The majority of cancer cases exhibit a significant presence of tumor-associated macrophages (TAMs). Tumor-promoting factors have been observed to exert an influence on the growth of tumors and the infiltration of lymphocytes, resulting in a state of immunosuppression (92). The utilization of M2-like macrophage-targeting nanoparticles, specifically denoted as PNP@R@M-T, has demonstrated notable efficacy in the targeted delivery of drugs to M1/M2-like macrophages and dendritic cells. This targeted drug delivery approach has been found to effectively reduce tumor size by 82% and significantly extend overall survival (92). In contemporary times, nanocarriers have developed as an innovative approach for the delivery of vaccinations (93). In order to enhance the therapeutic efficacy of nano vaccines in the context of cancer therapy, it is imperative to focus on DC targeting by means of modulating the structural characteristics of the vaccines (94). Zhang et al. have developed a personalized nano vaccine known as nano DC, which mimics the function of DCs, with the aim of stimulating T cell populations specific to tumor-associated antigens (TAAs) (95). Furthermore, Li, Y., et al. developed an *in-situ* vaccine formulation comprising polydopamine (PDA) nanoparticles coated with acid-responsive liposomes. The process of liposome catabolism, which specifically targets the tumor site, coupled with the utilization of PDA nanoparticles to facilitate photothermal therapy, synergistically promotes dendritic cell maturation and significantly enhances the impact on cytotoxic T-lymphocytes (96). Therefore, *in-situ* vaccines present a promising approach to enhance the effectiveness of cancer immunotherapy.

#### 4.2.5 Immune checkpoint inhibitor

The utilization of immune checkpoint inhibitor therapy is a nascent approach in the field of anti-tumor treatment, which aims to render cancer cells susceptible to the host immune system’s assault by selectively targeting particular molecules within the immune system. The efficacy of this treatment has been demonstrated across various tumor types, encompassing prostate cancer, lung cancer, melanoma, and other malignancies (97–100). Cytotoxic T-lymphocyte-associated protein 4 (CTLA-4) and programmed death-1 (PD-1) are recognized as checkpoints that impede the function of T cells (101).. Recent studies indicate that the use of T cell-targeted checkpoint antibodies, specifically anti-PD-1 or programmed death-ligand-1 (PD-L1), may modulate the functioning of innate immunity through both direct and indirect pathways, potentially influencing the overall effectiveness of clinical treatments (102). Wang, X., et al. suggested that the combination of an immune checkpoint inhibitor called anti-PD-L1 antibody and hollow mesoporous silica (HMS) nanospheres tumor vaccination can substantially enhance the population of CD4+ and CD8+ T cells. This, in turn, leads to an improvement in the effectiveness of immune checkpoint inhibitors as a treatment (103). Recently, a number of preclinical investigations have demonstrated that the efficacy of CTLA-4 antibody drugs relies on the elimination of Treg cells within the tumor, facilitated by the antibody’s heavy chain constant region Fc and immune cell Fc receptors (104, 105). In addition, Sato, Y., et al.,identified the contribution of the Fc-independent function of anti-CTLA-4 antibodies to anti-tumor effects (106). The approval of the immune checkpoint blocking (ICB) drug ipilimumab has significantly revitalized the field of cancer immunotherapy. ICB drugs function by inhibiting the interaction between receptors and ligands that are implicated in the pathway responsible for suppressing T-cell activation. Additionally, they hinder or reverse the development of acquired peripheral tolerance towards tumor antigens (107).

## 5 Conclusions

This bibliometric review examines the research on vaccines targeting TME using a bibliometric review. It analyzes worldwide collaborations, publications, and research hotspots in this field. These findings empower the scientific community to discern innovative concepts and pathways that will propel future tumor vaccination research. Due to the growing focus on studying the tumor microenvironment, there has been a rise in the development of vaccines for TME therapy. Nevertheless, there remain several pressing issues that need to be addressed in tumor vaccines utilizing TME. These include: 1. Finding solutions to combat the difficulties presented by tumor heterogeneity and immune escape mechanisms; 2. Overcoming the immunosuppressive TME that hampers the clinical efficacy of DC vaccines. 3. The majority of current studies primarily depend on therapeutic experiments conducted in live tumor mouse models that are genetically identical. These models cannot fully replicate the complex diversity and microenvironment of tumors in real patients. 4. How can we overcome the significant restriction of T cells caused by molecules like PD-1 and its binding partners PD-L1, CTLA-4, and LAG-3? 5. Exploring the complex mechanisms of tumor-tumor microenvironment interactions is critical for tumor vaccine development. Notwithstanding these issues, it is widely considered that the utilization of vaccines that target TME can be advanced by the integration of physiology, immunology, and chemistry in future research.

### 5.1 Limitations of the study

This study shows that there are a few problems. First, the vaccines for TME studies were taken from a single database (WOSCC) so that they would fit the data format for bibliometric tools in both VOSviewer and CiteSpace. This could have led to selection bias. There are also other sources of data, like PubMed or Scopus, but most of them only work with one of the bibliometric tools, usually VOSviewer. So, we decided to use two bibliometric tools (CiteSpace and VOSviewer) to reduce selection bias and get rid of the hassle of putting together similar literature from different sources.

Furthermore, the present study may be subject to language bias as it exclusively incorporated articles published in the English language. In order to achieve comprehensive findings, future investigations should consider the inclusion of publications in languages other than the predominant one.

## Author contributions

YL: Data curation, Formal Analysis, Methodology, Writing – original draft. SL: Data curation, Methodology, Visualization, Writing – original draft. LC: Writing – original draft. LL: Writing – original draft. CX: Writing – original draft. HQ: Writing – original draft. XL: Writing – original draft. HC: Funding acquisition, Supervision, Writing – review & editing. KL: Supervision, Writing – review & editing.
