# Supplementary material for: Global trends in tumor microenvironment-related research on tumor vaccine: a review and bibliometric analysis
Source: Front Immunol. 2024 Feb 6;15:1341596. doi: 10.3389/fimmu.2024.1341596 (PMC10876793; doi:10.3389/fimmu.2024.1341596)
Supplement: Supplementary file 1 [file Table_1.docx]

Table S1. Thesaurus

| label | replace by |
| --- | --- |
| 1st-line treatment | 1st-line therapy |
| agonists | agonist |
| anti-pd1 | anti-pd-1 |
| antibodies | antibody |
| antigen presentation | antigen delivery |
| antigen-presenting cells | antigen-presenting cell |
| antitumor immune-responses | antitumor immune-response |
| antitumor responses | antitumor response |
| b cells | b-cell |
| b-cells | b-cell |
| biomarkers | biomarker |
| bone-marrow | bone marrow |
| breast cancer | breast neoplasms |
| breast-cancer | breast neoplasms |
| cancer stem cells | cancer stem cell |
| cancer stem-cells | cancer stem cell |
| cancer vaccines | cancer vaccine |
| cancer-associated fibroblasts | cancer-associated fibroblast |
| cancer/testis antigens | cancer-testis antigen |
| car-t | car t |
| car-t-cells | car t cells |
| cd8 t-cells | cd8 t cells |
| cd8(+) t cells | cd8(+) t cell |
| cd8(+) t-cell | cd8(+) t cell |
| cd8(+) t-cells | cd8(+) t cell |
| central-nervous-system | central nervous system |
| cervical-cancer | cervical cancer |
| checkpoint blockade | checkpoint blockade |
| checkpoint inhibition | checkpoint inhibitor |
| checkpoint inhibitors | checkpoint inhibitor |
| chemokines | chemokine |
| chimeric antigen receptors | chimeric antigen receptor |
| clinical trials | clinical trial |
| clinical-trial | clinical trial |
| clinical-trials | clinical trial |
| colon-cancer | colon cancer |
| colorectal-cancer | colorectal cancer |
| combination therapies | combination therapy |
| cytokines | cytokine |
| cytolytic t-lymphocytes | cytotoxic t lymphocytes |
| cytotoxic t-lymphocytes | cytotoxic t lymphocytes |
| dendritic cell (dc) | dendritic cell |
| dendritic cell vaccines | dendritic cell vaccine |
| dendritic cells | dendritic cell |
| dendritic cells (dcs) | dendritic cell |
| dna vaccines | dna vaccine |
| drug-delivery | drug delivery |
| drug-resistance | drug resistance |
| endothelial-growth-factor | endothelial growth-factor |
| epitopes | epitope |
| epstein-barr-virus | epstein-barr virus |
| exosomes | exosome |
| gastric-cancer | gastric cancer |
| gene-therapy | gene therapy |
| glioblastoma-multiforme | glioblastoma multiforme |
| hepatocellular-carcinoma | hepatocellular carcinoma |
| high-grade gliomas | high-grade glioma |
| immune checkpoint blockade | immune checkpoint inhibitor |
| immune checkpoint inhibition | immune checkpoint inhibitor |
| immune checkpoint inhibitorabbreviations | immune checkpoint inhibitor |
| immune checkpoint inhibitors | immune checkpoint inhibitor |
| immune checkpoints | immune checkpoint |
| immune evasion | immune escape |
| immune-checkpoint blockade | immune checkpoint inhibitor |
| immune-responses | immune-response |
| immunogenic cell-death | immunogenic cell death |
| immunomodulation | immune modulation |
| immunosuppression | immune suppression |
| immunosurveillance | immune surveillance |
| immunotherapies | immune therapy |
| immunotherapy | immune therapy |
| in-situ vaccination | in situ vaccination |
| inhibitors | inhibitor |
| liposomes | liposome |
| lung-cancer | lung cancer |
| lymph-nodes | lymph-node |
| macrophages | macrophage |
| malignant gliomas | malignant glioma |
| mechanisms | mechanism |
| mhc class-i | mhc class i |
| micrornas | microrna |
| models | model |
| monoclonal antibodies | monoclonal antibody |
| monoclonal-antibodies | monoclonal antibody |
| monoclonal-antibody | monoclonal antibody |
| mrna vaccines | mrna vaccine |
| multiple-myeloma | multiple myeloma |
| myeloid suppressor-cells | myeloid-derived suppressor cell |
| myeloid-derived suppressor cells | myeloid-derived suppressor cell |
| nanoparticles | nanoparticle |
| nanovaccines | nanovaccine |
| natural killer | natural killer cell |
| natural killer cell | natural killer cell |
| natural killer cells | natural killer cell |
| natural-killer-cells | natural killer cell |
| neoantigens | neoantigen |
| oncolytic viruses | oncolytic virus |
| ovarian-cancer | ovarian cancer |
| pancreatic-cancer | pancreatic cancer |
| pattern-recognition receptors | pattern recognition receptors |
| peptide vaccines | peptide vaccine |
| programmed death-1 | programmed death 1 |
| prostate-cancer | prostate cancer |
| radiation-therapy | radiation therapy |
| receptors | receptor |
| regulatory t | regulatory t cells |
| regulatory t cells (tregs) | regulatory t cells |
| regulatory t-cells | regulatory t cells |
| renal-cell carcinoma | renal cell carcinoma |
| signaling pathways | signaling pathway |
| t cells | t cell |
| t-cell | t cell |
| t-cells | t cell |
| t-lymphocytes | t lymphocytes |
| targeted therapies | targeted therapy |
| tertiary lymphoid structures | tertiary lymphoid structure |
| tgf-beta | tgf beta |
| therapeutic cancer vaccines | therapeutic cancer vaccine |
| therapeutic vaccines | therapeutic vaccine |
| toll-like receptors | toll-like receptor |
| tumor antigens | tumor antigen |
| tumor microenvironment (tme) | tumor microenvironment |
| tumor vaccines | tumor vaccine |
| tumor-antigen | tumor antigen |
| tumor-antigens | tumor antigen |
| tumor-associated antigens | tumor-associated antigen |
| tumor-associated macrophages | tumor-associated macrophage |
| tumors | tumor |
| tumour immunotherapy | tumor immunotherapy |
| tumour microenvironment | tumor microenvironment |
| tyrosine kinase inhibitors | tyrosine kinase inhibitor |
| vaccines | vaccine |
| viruses | virus |
